# Letter from the Editor in Chief

**DOI:** 10.19102/icrm.2019.100606

**Published:** 2019-06-15

**Authors:** Moussa Mansour


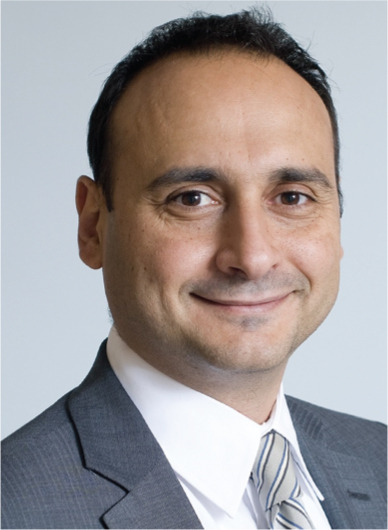


Dear Readers,

During the annual scientific meeting of the Heart Rhythm Society last month in San Francisco, CA, a number of important scientific studies were presented, including especially during the late-breaking clinical trials sessions. Here, I would like to highlight three that are likely to have a significant impact in the area of atrial fibrillation (AF) ablation.

The first is the Evaluate Renal Artery Denervation in Addition to Catheter Ablation to Eliminate AF (ERADICATE-AF) trial. Conducted in Europe, this multicenter study enrolled patients with paroxysmal AF and hypertension who were randomized to receive either pulmonary vein (PV) isolation (PVI) alone or PVI plus renal denervation. The primary endpoint of the study, which was freedom from AF, was significantly improved with the hybrid procedure in comparison with PVI alone (72.1% versus 56.5%; p = 0.004). Furthermore, the addition of renal denervation did not seem to increase the complication rate of the procedure, which was found to be about 4% to 5% in both arms.

The second study of note was the Real-time Electrogram Analysis for Drivers of AF (RADAR) trial. This first-in-human multicenter, single-arm, United States Food and Drug Administration Investigational Device Exemption trial evaluated a novel system designed to identify drivers for AF in patients with the persistent form of this disease. Using a circular multielectrode catheter in the left atrium, repetitive patterns of activation in the coronary sinus were sequentially mapped and stitched together to generate panoramic displays. These activation maps were combined with voltage maps to highlight areas with high probabilities of driving AF. When the drivers were ablated in addition to performing PVI, 72% of patients remained free from all atrial arrhythmias and 83% remained free from AF and off antiarrhythmic medications at nine months ± three months of follow-up.

Third was the Pulsed-field Ablation for PVI study, which investigated the lesion durability and long-term safety associated with pulsed-field ablation incorporated during PVI in patients with AF. Three months after the index procedure, the patients were remapped to assess for PV reconnection. The results were very impressive, revealing findings of ultra-rapid PVI with 0% adenosine reconnection, 100% durable PVI at three months, and 87.1% ± 5.6% freedom from AF at 12 months of follow-up, respectively. In addition, there were no strokes or transient ischemic attacks, phrenic injury, PV stenosis, or esophageal injury.

The above three studies seek to fulfill two unmet needs in the ablation of AF, as follows: (1) the need for fast and durable PVI and (2) the identification of adjunct targets in addition to PVI in patients with persistent AF. The preliminary findings from these trials are very promising, though larger studies are needed to confirm the early findings. I believe that these three technologies will ultimately have a significant impact in the ablation of AF.

I hope that you enjoy reading this issue of the journal and that you find its content of educational value.

Sincerely,


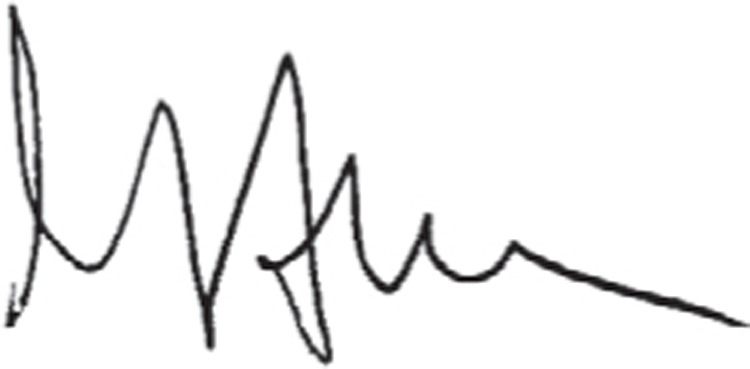


Moussa Mansour, MD, FHRS, FACC

Editor in Chief

The Journal of Innovations in Cardiac Rhythm Management

MMansour@InnovationsInCRM.com

Director, Atrial Fibrillation Program

Jeremy Ruskin and Dan Starks Endowed Chair in Cardiology

Massachusetts General Hospital

Boston, MA 02114

